# Colony Site Selection of Gray Heron (*Ardea cinerea*) During the Breeding Period at Multiple Spatial Scales

**DOI:** 10.1002/ece3.70937

**Published:** 2025-02-12

**Authors:** Ran Tian, Donghong Li, Shiyu Zhang, Yongbin Zhao, Guodong Yi

**Affiliations:** ^1^ School of Life Sciences Jilin Normal University Siping Jilin China

**Keywords:** colony site selection, cross‐regional, gray heron, multiple spatial scales

## Abstract

The colony site selection of birds reflects their adaptability to the ecological environment. As one of the most common and widely distributed heron species, the gray heron ( *Ardea cinerea* ) serves as an interesting study case for ornithologists. Researchers often study their colony site selection characteristics to understand how they adapt to different ecological environments and how these adaptation strategies affect their survival and reproduction. However, the majority of research has focused solely on studying the gray heron in a single region at scales. To maintain the model's generalization ability and ensure accurate predictions of gray heron colony preferences, we avoided using excessively similar landscapes within a single landscape mode. This study utilizes geographic information systems (GIS) and random forest (RM) models to examine the colony site selection during the breeding period of gray herons across various regions and spatial scales, providing insights into their adaptability and the environmental factors influencing their colony site selection. By conducting research across most regions of China, we gain valuable insights into gray heron adaptability and colony strategies across various environments. The results indicate that potentially suitable foraging habitats are the primary determinant of gray heron colony site selection. When habitat requirements are met, gray heron exhibit a degree of flexibility in colony site choice, highlighting their adaptive behaviors and potentially offering new insights into their widespread distribution. By employing this approach, our findings offer new insights into wildlife conservation, emphasizing the importance of interdisciplinary collaboration in shaping conservation strategies. Additionally, the methods used in this study may be applicable to other bird species and colony groups, providing valuable insights into habitat preferences across diverse ecological contexts.

## Graphical abstract

Exploring nest site selection of herons in multiple regions and scales during brooding period through random forest model and GIS.

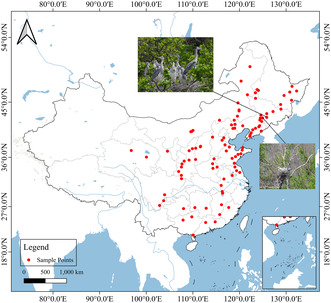

## 1 Introduction

Colony site selection is a crucial component of the life history of colonial birds, serving not only as a vital environmental requirement for individuals, populations, or communities to complete specific life stages (such as the brooding period) but also as a key factor in ensuring successful reproduction and meeting basic survival needs such as food, water, concealment, and safety (Healy, Tello‐Ramos, and Hébert 2023). The choice of colony site directly impacts bird habits, reproduction, and chick rearing (Carroll et al. 2015; Hennicke and Weimerskirch 2014). Increasing research indicates that birds follow specific criteria for colony site selection, with different species exhibiting unique preferences, including the size and density of the colony (Tong et al. 2021). Increasing research indicates that birds follow specific criteria for colony site selection, with different species exhibiting unique preferences, including the size and density of the colony (Brown, Stutchbury, and Walsh 1990). While the specific factors influencing bird colony site selection have been extensively studied since the 1970s, the interplay between these factors remains complex. While it is widely recognized that environmental factors such as food resources, nesting materials, predation risks, and climatic conditions influence bird choices (Di Sallo and Cockle 2022), the specific dynamics and interactions of these factors in shaping colony site selection remain inadequately explored. Addressing this gap is crucial for understanding how these influences vary across different ecological contexts and could lead to new insights into avian adaptability and survival strategies (Thompson 2007).

Most current research on bird colony site selection focuses on a single region, encompassing various bird species, and cross‐regional studies are relatively scarce (Gabel, Frederick, and Zabala 2021). The methodologies applied in single‐region studies are particularly effective when investigating endangered bird species because they often have highly consistent colony site requirements, which contributes to their endangered status (Di Sallo and Cockle 2022; Swaisgood et al. 2018). However, for birds with a wider distribution and larger populations and that are capable of adapting to diverse and changing environments, there could be significant differences in cross‐regional colony site preferences (Carrasco, Toquenaga, and Mashiko 2017). However, for birds with a wider distribution and larger populations and that are capable of adapting to diverse and changing environments, there could be significant differences in cross‐regional colony site preferences (Carrasco, Toquenaga, and Mashiko 2017). Hence, cross‐regional studies are crucial for obtaining a comprehensive understanding of these birds, especially large waterbirds located at the top of the food chain, as their ability to habituate to human disturbances plays a significant role in their survival and distribution (Momberg et al. 2023). Moreover, the application of global positioning system (GPS) technology has shown that large waterbirds typically forage within a 3–5 km radius around their colonies during the breeding period (Momberg et al. 2023; Webster and Cardina 1997). Therefore, large‐scale habitat features within a 3–5 km radius of their colony sites could significantly influence them (Momberg et al. 2023). Consequently, the study of large waterbird colony site selection should also be extended to multiple spatial scales.

It should be emphasized that the gray heron ( *Ardea cinerea* ), a large waterbird in the family Ardeidae, is exceedingly common on the continents of Europe and Africa and is widely distributed in China according to data from the Global Biodiversity Information Facility (GBIF) [http://www.gbif.org/] (Forti et al. 2023; Gustinelli et al. 2010). Understanding its colony site selection is crucial, as it reflects broader ecological dynamics and adaptation strategies, particularly in response to environmental changes.

Early studies on colony site selection by gray heron primarily focused on habitat characteristics at a single spatial scale around colony sites. Recently, an increasing number of studies have explored gray heron colony site selection at multiple spatial scales (Carrasco, Mashiko, and Toquenaga 2014; Kelly et al. 2008; Manikowska‐Ślepowrońska et al. 2016). Methodologically, as spatial complexity increases, particularly in agricultural landscapes influenced by urbanization, the optimization problem in ecological models becomes more complex (Cannon et al. 2020; Warren et al. 2014). This complexity has rendered traditional linear methods ineffective in handling the intricate relationships between habitat variables and community locations (Warren et al. 2014). Consequently, most of these studies have abandoned the traditional linear response assumption, turning instead to machine learning methods such as support vector machines (SVM), decision trees (DM), and random forest (RM) algorithms. Nevertheless, the majority of these studies are confined to single regions. By limiting the scope of research to smaller geographical areas, buffer zones around sample points may exhibit excessive overlap or similarity, leading to a high degree of homogeneity in landscape features among samples (Dindaroğlu et al. 2015; Vandermeer 2020). This homogeneity weakens the robustness of the models and limits their predictive power in new areas. As the gray heron has a wide distribution across Europe and Asia (Cannon et al. 2020; Mechenich and Žliobaitė 2023), research on its colony site selection needs to transcend the limitations of single‐region studies and simplistic linear models (Giammarino, Quatto, and Renna 2021). By adopting cross‐regional, multiple spatial scale modeling, the aim is to enhance the generalizability and applicability of these studies (Piano et al. 2020). This approach captures the complexity of ecosystems more comprehensively, thereby more accurately predicting colony site selection (Momberg et al. 2023).

Although gray herons are generally considered sensitive to human disturbances at their colony sites, they are among the most adaptable species of herons worldwide. They can colony in a variety of environments, including roadsides, fuel stations, and public gardens. The small sample size used in species habitat model exercise may prevent the disclosure of this variability (Manikowska‐Ślepowrońska et al. 2015). However, the issue of sensitivity to human disturbance is complex and likely varies in relation to human attitudes. In regions where gray herons are not persecuted, they may colonize even inside human settlements (Bhatnagar and Shekhawat 2014; Trivedi and Parasharya 2019). Conversely, in other regions, human presence is avoided, and colony is restricted to sites protected by water bodies. This variability suggests that gray herons exhibit a certain flexibility in their colony site selection. They may not follow fixed criteria; instead, they consider a balance of various factors such as food resources, nesting materials, and the degree of human tolerance. Consequently, gray herons might tolerate some unfavorable conditions if the benefits from human activities outweigh the drawbacks. If human activities slightly impact their habitat and reproduction but do not pose a direct threat, gray herons may choose to remain near areas of human activity, reflecting this balance in their site selection (Jakubas and Manikowska‐Slepowronska 2013). Finally, landscape diversity significantly influences gray heron colony site selection. They may prefer complex and variable landscape environments. To test these hypotheses, this study employs geographic information system (GIS) technology and RM models to quantitatively and accurately assess the impact of land‐use changes on gray heron colony site selection during the breeding period across different regions and multiple spatial scales. This approach aims to provide a new perspective for understanding colony site selection in gray herons.

## 2 Materials and Methods

### 2.1 Study Area

#### 2.1.1 Collection of Gray Heron Colony Coordinates

**FIGURE 1:**
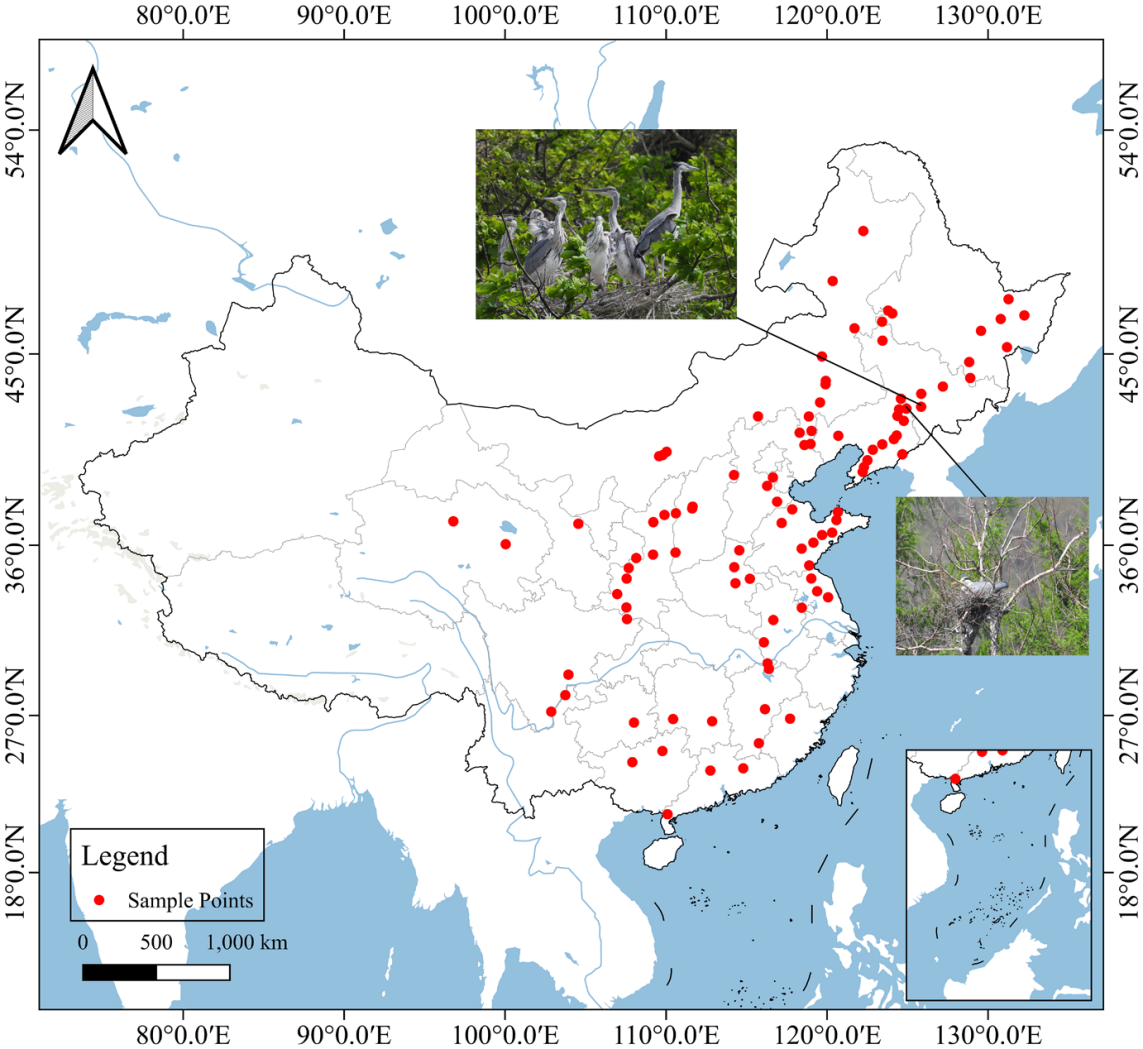
The 100 sample points of heron nest‐site in study areas.

To ensure sufficient variability in the landscapes studied, a principle was established for this research: data collection did not include colony sites located within 100 km of each other. By systematically reviewing related papers from Chinese academic databases such as CNKI, cqVIP, and Wanfang published within between 2018 and 2023, we successfully identified 19 coordinates of confirmed gray heron colony sites located in China. Additionally, field investigations were conducted in Yehetown, Siping city, Jilin Province, and Bailuzhou Tourism Resort, Kaiyuan city, Liaoning Province. Including the 79 colony sites obtained from the GBIF website, a total of 100 sampling sites were identified (Locations where both gray herons and colonies appear in the same photograph are considered Gray heron colony sites.). All these data were collected during the breeding season of the Gray heron (April to June) (Figure 1), encompassing tree nests, ground nests, and artificial nests. These colony sites feature a variety of habitat types, including forests, wetlands, and deserts, and exhibit significant differences in their proximity to human activities. Some of these colonies are located in natural reserves with minimal human activity, while others are located near villages where human activities are more frequent. Nonmetric multidimensional scaling (NMDS) analysis was then used to test whether sampling points were clustered to assess landscape diversity (Calbi et al. 2021; Miranda‐Gallegos et al. 2023).

#### 2.1.2 Collection of Pseudoabsence Points

To further explore the differences between the areas surrounding gray heron colonies and unoccupied areas, pseudoabsence points were collected as negative samples. Preliminary data collection was conducted on the GBIF platform, from which 22,541 coordinates of gray heron sightings were gathered. After filtering out records that were too close together or that had incomplete latitude and longitude information, 21,581 valid coordinates were obtained. Subsequently, Python was used for sampling pseudoabsence points. Assuming these 21,581 coordinates all represented gray heron colonies, pseudoabsence points were generated outside a 5 km radius of these coordinates. To ensure the completeness of the land use information, the generated pseudoabsence points were set to be more than 100 km away from the national borders, and large towns were avoided. It should be noted that, as field verification of these randomly generated coordinates was not possible, it cannot be entirely confirmed whether these pseudoabsence points are actually gray heron colony sites. To avoid potential dataset imbalances that might arise from the random sampling process, 500 random coordinates were generated, and detailed statistics on the land use around these coordinates were compiled. Then, 100 of these 500 coordinates were randomly selected as negative samples to form a community along with the previously collected data. Multiple iterations of calculation with replacement were conducted to balance potential errors (Ramya and Ganapathy 2022).

#### 2.1.3 Environmental Variables

The analysis of land use around gray heron colony sites utilized 2021 land use grid data (with a 30 m resolution) from the Earth System Science Data Database to depict the surrounding environment of the colonies in detail (Yang and Huang 2021). The land use information primarily included the following eight variables: cropland, forest, shrub, grassland, water, barren, impervious, and wetland (Table 1). Using ArcGIS software (version 10.8, Esri, Redlands, CA, USA), multilayer circular buffer zones were established around the 100 gray heron sample points and 100 randomly generated negative sample points. Within each buffer zone, the areas of these eight types of land use variables were calculated. The radii of the buffer zones were set at 0.5, 1, 2, 3, 4, 5, 6, 7, 8, 9, 10, 15 and 20 km, covering land use situations from near to far at different scales. Although RM models can handle interactions and nonlinear effects between variables and adapt to the influence of spatial autocorrelation (Georganos et al. 2021; Song et al. 2017), Pearson correlation coefficients were used to analyze the relationships between environmental variables at the scales of interest. This was done to further examine whether the model adequately considered spatial autocorrelation and to more comprehensively explain the interplay between variables at different scales. Finally, after completing the land use information statistics, R software (v 4.2.2) was used to calculate the Shannon diversity index of land use at different scale coordinates. This method allows for the quantification of the diversity of land use types at different scales, thereby assessing how these diversities impact the selection of gray heron colony sites (Bhatnagar and Shekhawat 2014; Hachour, Talmat‐Chaouchi, and Moulaï 2021).

**TABLE 1:** Land use information.

Variables	Definition
Cropland	Land primarily utilized for the cultivation and harvesting of crops
Forest	An area predominantly composed of trees, covering a large expanse
Shrub	Land areas constituted by bushes or low trees
Grassland	Regions mainly comprised of herbaceous plants, often utilized for grazing and grassland ecosystem research
Water	Areas encompassing rivers, lakes, reservoirs, and other significant bodies of water
Barren	Land with low soil fertility, sparse or no vegetation cover
Impervious	Impermeable cover (such as impermeable road surface, roof)
Wetland	Areas where the land is regularly or seasonally saturated with water or where the soil remains waterlogged

### 2.2 Data Analysis

#### 2.2.1 RM Model

The RM algorithm was used to establish a gray heron colony site selection model for analyzing and predicting the geographical distribution of colonies. This study utilized 100 gray heron colony sample points observed in the field, along with 100 hypothetical nonexistent colony points generated through random sampling. The land use types around each sample point were precisely quantified to form the input dataset. To construct a more accurate model, RM models at different scales were created, with each model utilizing land use information within different buffer zones. For instance, in the 3 km buffer zone model, only the land use variables within that buffer zone were considered. This approach aims to capture the impact of land use types on gray heron colony site selection at different spatial scales. To validate the predictive capability of the model and ensure the randomness of the training set data, thereby reducing errors due to sample selection bias and ensuring the model's predictive accuracy across different datasets, each RM model used 30% of the samples as out‐of‐bag (OOB) data. In each classification model, 500 trees were built, and the dataset of 500 hypothetical nonexistent points underwent bootstrap resampling (Ljumovic and Klar 2015).

#### 2.2.2 Predictive Accuracy

In the RM models used in this study, each decision tree was constructed based on bootstrap sampling of the dataset, with approximately 30% of the samples left out and not used in building the corresponding tree. These left‐out samples are known as OOB samples, providing an in‐built cross‐validation mechanism for the RM. Hence, there is no need for additional cross‐validation to obtain an unbiased estimate of the test set error (Huang et al. 2020). Since the RM algorithm uses the 30% of the data remaining in the bootstrap sample of a particular tree for classification, approximately 20% of the trees are used to test all the data. The proportion of classifications that do not match the true categories represents the OOB error estimate. Additionally, due to the inherent uncertainty of random sampling, even with the same seed and parameters, the results may vary slightly (Bitner‐Gregersen et al. 2020). To address this, 100 models were constructed at each scale, and the average of the 100 OOB error values was used to measure the model's accuracy. Variations in predictive accuracy were observed among different random sets of unselected sites. These random sets are somewhat analogous to datasets used for supervised learning in neural network models (Ching, Zhu, and Garmire 2018). Potential differences between data may lead to increased accuracy errors. Therefore, the average accuracy of the 100 modeling iterations was calculated, but the highest accuracy observed at each scale was also a focus. While average accuracy reflects the overall trend of model performance across scales, to accurately assess the best performance of the RM model at each scale, the maximum accuracy value must be considered (Yan et al. 2023). This is because the goal is to use the dataset that best explains the population distribution rather than merely focusing on an ‘average model’ that provides an average explanation.

#### 2.2.3 Variation in Accuracy

To verify the presence of outliers in the sample points, random data deletion was performed when establishing new models. By repeatedly executing this process, the average accuracy after each modeling iteration was calculated, and the presence of outliers in the sample was assessed (Haidar and Gaber 2017; Kuhlmann et al. 2023). Since the randomly deleted data might not all be outliers, the number of deletions determined the repetitions to ensure that each dataset was considered a potential outlier, identifying the group with the highest average accuracy. The significance of the number of deleted samples was also considered. Excessive data deletion may result in the new dataset yielding results that are entirely inconsistent with those of the original dataset. Therefore, the number of deletions was limited, and five iterations were performed, ranging from deleting one data point to a maximum of 50 (García‐Pedrajas and Ortiz‐Boyer 2008). By comparing the accuracy of models with undeleted data, the presence of outliers in the dataset can be assessed (Kuhlmann et al. 2023).

### 2.3 Shannon Index

The Shannon diversity index of the landscape was calculated for different scales of collection points and pseudoabsence points. For spatial scale data that conformed to a normal distribution, a t test was used to determine whether there were significant differences in the Shannon index between the sampling points and the pseudoabsence points. For data not fitting a normal distribution, the Mann–Whitney U test was employed.

## 3 Results

### 3.1 Landscape Diversity of the Sampling Points

**FIGURE 2:**
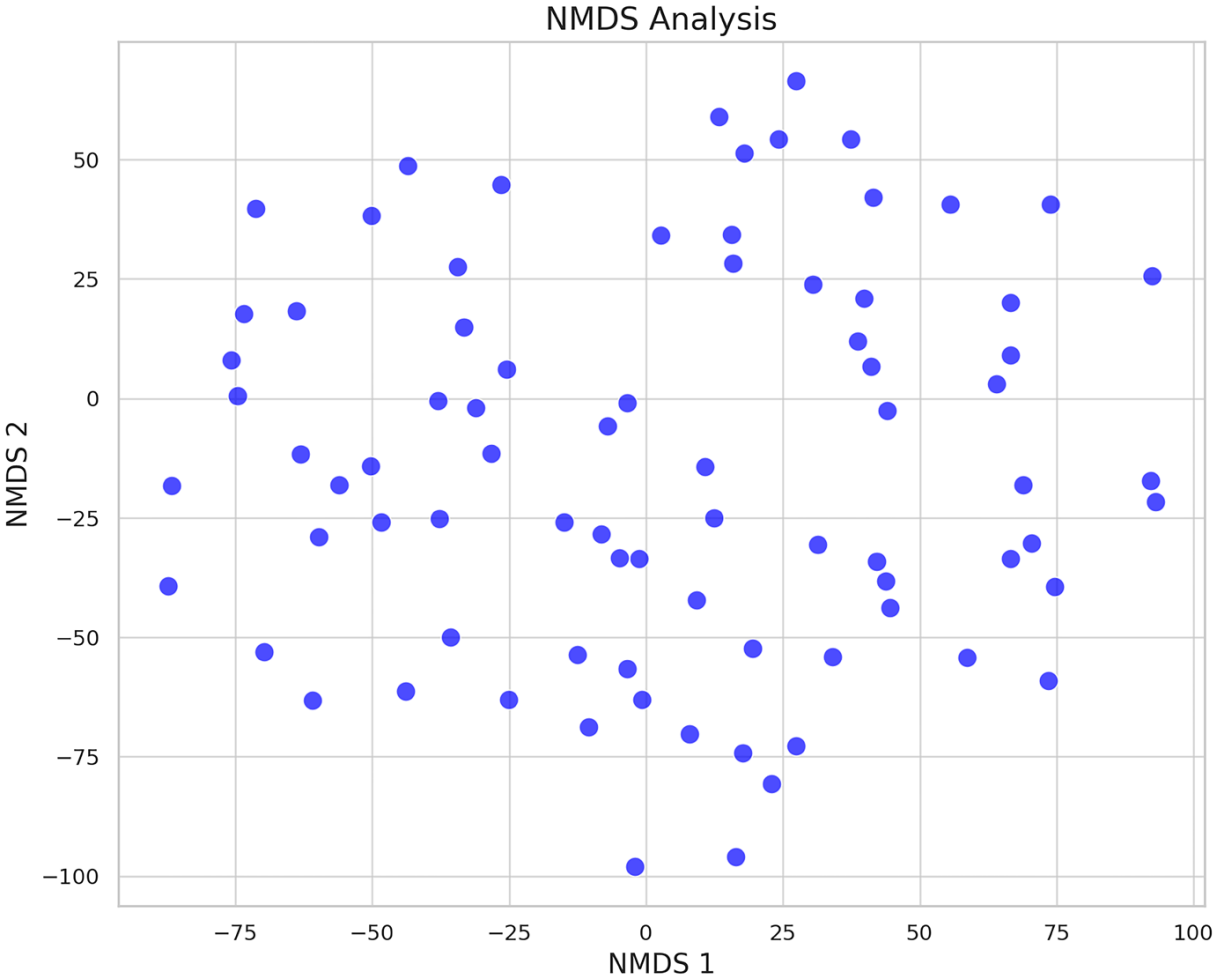
Nonmetric multidimensional scaling (NMDS) image showing no discernible clustering among the sampling points.

NMDS analysis of our land use data revealed significant variations in spatial characteristics across the sampled sites (stress = 0.152, *p* < 0.05). The NMDS stress value indicates a good fit, suggesting that the 2D representation reliably reflects the complex relationships in our multidimensional data. This analysis highlighted nuanced differences between sites, which were not as evident in traditional cluster analysis, indicating a more intricate pattern of land use. The lack of distinct clustering in the NMDS plot (Figure 2) suggests overlapping or shared land use features among the sites, revealing a landscape with complex ecological interactions.

### 3.2 Model Results

**FIGURE 3:**
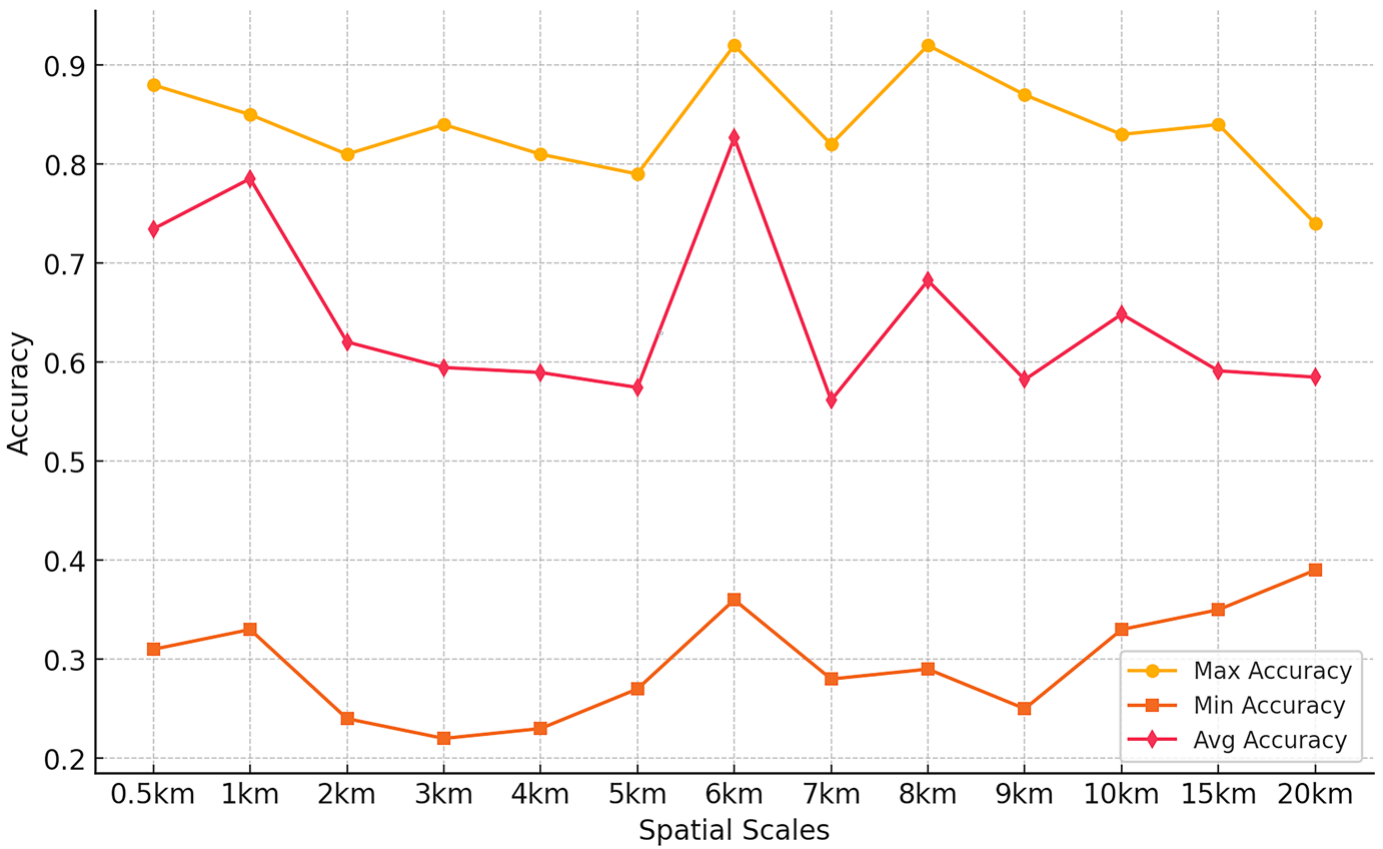
The maximum, average, and minimum accuracy of the random forest model at various spatial scales.

The accuracy of the RM model is influenced by several factors, including sample size, feature selection, and parameter settings. Despite these influences, the results show that the accuracy of the models across all spatial scales generally ranges from 22% to 92%, the average accuracy remains between 56% and 82% (Figure 3). Among these models, the 1 km scale model exhibited the highest average accuracy, reaching 82%, followed by the 0.5 and 6 km scales. Additionally, including several scales with higher average accuracy, the maximum accuracies at the 4, 15, and 20 km scales also reached 81%, while the minimum accuracies for these scales were 23%, 35%, and 39%, respectively. However, their average accuracies were not as strong. Therefore, apart from the 0.5, 1, and 6 km scales, other scales are not considered characteristic for explaining colony site selection. Pearson's coefficient indicates moderate correlations between land variables (Tables 2, 3, 4).

**TABLE 2:** Pearson's correlation coefficient between the land use variables among all the buffers at the 0.5 km scale.

Land use	Forest	Shrub	Grassland	Water	Barren	Impervious	Wetland
Cropland	−0.38235	−0.00521	−0.48353	−0.08599	−0.24813	−0.25437	−0.25865
Forest		−0.08282	0.21024	0.000854	−0.12695	0.25589	−0.05287
Shrub			0.152832	−0.04725	0.05748	−0.09525	0.47855
Grassland				−0.22752	0.0911	0.15829	−0.05621
Water					−0.0658	0.01725	0.14526
Barren						−0.0145	0.48268
Impervious							−0.41565

**TABLE 3:** Pearson's correlation coefficient between land use variables among all buffers at the 1‐km scale.

Land use	Forest	Shrub	Grassland	Water	Barren	Impervious	Wetland
Cropland	−0.28013	−0.04603	−0.27807	−0.12257	−0.11422	−0.13219	−0.1025
Forest		−0.07591	−0.16503	0.004786	−0.09449	−0.22581	0.05664
Shrub			0.20425	−0.04303	0.01581	0.07523	0.01847
Grassland				−0.07124	−0.08236	0.15764	−0.06848
Water					−0.05563	0.018164	0.02608
Barren						−0.02889	0.42561
Impervious							−0.06827

**TABLE 4:** Pearson's correlation coefficient between land use variables among all buffers at the 6‐km scale.

Land use	Forest	Shrub	Grassland	Water	Barren	Impervious	Wetland
Cropland	−0.34528	−0.09449	0.354827	0.009538	−0. 0649	0.183435	−0.4528
Forest		0.04539	−0.153613	0.093811	−0.08495	−0.268433	−0.35869
Shrub			0.045892	−0.06484	0.03874	0.36458	−0.07528
Grassland				−0.1154	−0.0548	0.08544	0.0058
Water					−0.0568	0.153617	0.01876
Barren						−0.15846	−0.73632
Impervious							−0.18231

**FIGURE 4:**
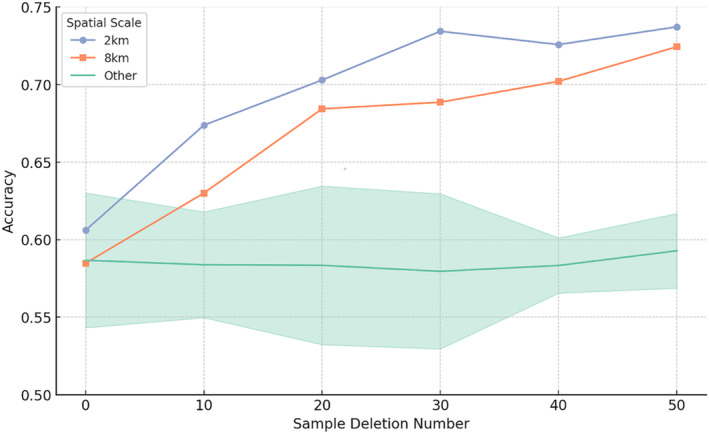
After randomly removing some samples from the random forest model, the accuracy increased at spatial scales of 2 and 8 km, while there was no significant change at other spatial scales. ‘Other’ refers to all spatial scales except 2 and 8 km.

**FIGURE 5:**
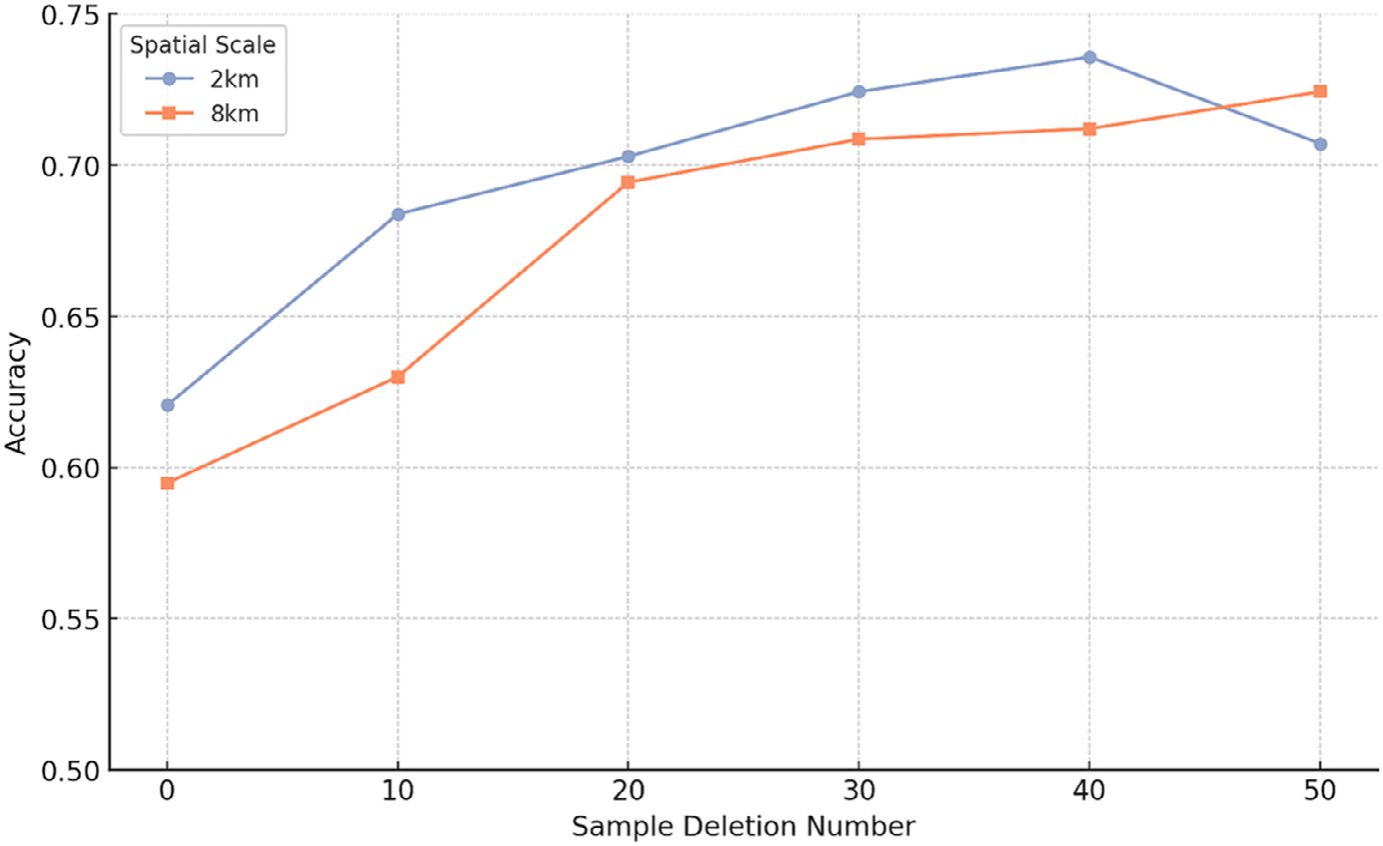
In the new model, there is an upward trend in accuracy at spatial scales of 2 and 8 km.

In the model, after removing certain sample data identified as outliers based on their significantly lower accuracy scores, there was a noticeable increase in average accuracy at the 2 km and 8 km scales, as indicated by linear regression (Figure 4). This suggests that the initial presence of these outliers negatively impacted the model's performance at these two scales. New calculations were conducted using annular scales formed by the neighboring scales with better models, specifically the 1–2 km and 6–8 km rings. The results show that as the number of deleted samples increases, so does the model's accuracy (Figure 5).

Other scales did not show significant changes in accuracy after the deletion of some samples, indicating a certain level of robustness in the model when processing data at these scales. In other words, the model shows a degree of flexibility across different sample combinations, indicating that it does not heavily depend on specific samples for its overall performance.

### 3.3 Variable Importance

**FIGURE 6:**
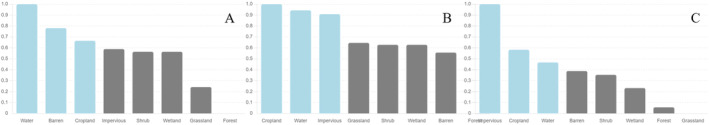
The relative importance of eight variables was investigated using a random forest model. The annotations ‘A’, ‘B’, and ‘C’ correspond to scales of 0.5, 1, and 6 km, respectively.

An importance analysis was performed on the highest accuracy models at the 0.5 km, 1 km, and 6 km scales. At the 0.5 km scale, water bodies were the most important variable for explaining colony site selection (Figure 6). The significance of water bodies fluctuated greatly across different spatial scales. Within the 0.5 km to 10 km range, the importance of water bodies ranked highly, but it was less significant at scales greater than 10 km. At the 1 km scale, grassland was identified as the most crucial variable. Across all scales, the significance of cropland consistently ranked high (Figure 6). At the 6 km scale, the importance of impervious surfaces was the highest, while at other scales, its importance was moderate (Figure 6; Additional files 1–10).

### 3.4 Shannon Index

**FIGURE 7:**
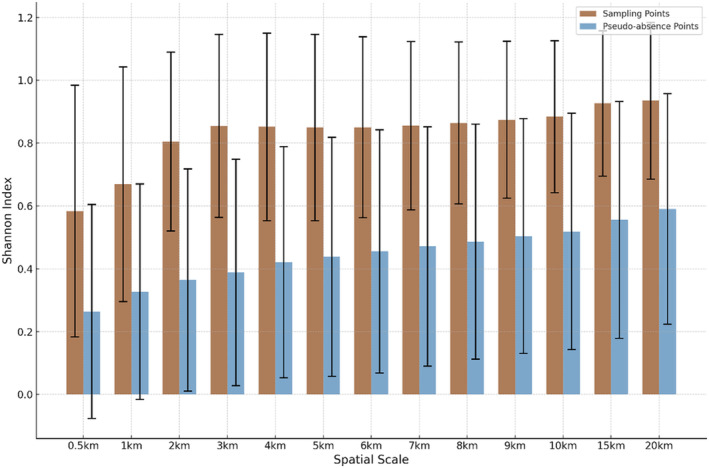
The Shannon index for sampling points and pseudorandom points across different scales.

The differences in the Shannon diversity index for both the collection points and pseudoabsence points at all the scales were highly significant (Figure 7). At any scale, gray herons showed a preference for areas with more complex landscapes. This significance is evident across all distance scales, with the Mann–Whitney *U* test results showing *p* values ranging from 0.012328 at 0.5 km to 0.000131 at 3 km, confirming the preference for complex landscapes at every examined scale (e.g., at 0.5 km, U = 320, *p* = 0.012; at 1 km, U = 336, *p* = 0.004; at 2 km, U = 362, *p* < 0.001; Table 5).

**TABLE 5:** The Mann–Whitney *U* test indicates significant differences between sample points and pseudoabsence points at various spatial scales.

Spatial scales	Statistic	*p*
0.5 km	323	0.005364
1 km	337	0.006684
2 km	352	0.000362
3 km	371	0.000094
4 km	354	0.000254
5 km	355	0.001365
6 km	385	0.001584
7 km	326.5	0.002583
8 km	336.5	0.003593
9 km	348.5	0.004458
10 km	339	0.001083
15 km	348	0.001251
20 km	345	0.003156

## 4 Discussion

### 4.1 Key Factors Influencing Colony Site Selection

This study revealed that at smaller scales, water bodies and cropland were identified as the most important variables at the 0.5 km and 1 km scales, respectively. However, it is crucial to note that the land use grid data did not distinguish between paddy fields, which can be used by foraging gray herons, and dry croplands, which are used infrequently. Based on the high demand for food resources during the breeding period, we expected that gray herons would forage intensively within 1 km of their colonies, our findings suggest otherwise; gray herons do not typically forage intensively in this range. This observation aligns with literature data indicating that, in certain contexts, colony safety may outweigh food availability (Jakubas and Manikowska‐Slepowronska 2013). Furthermore, choosing to reside near water bodies ensures that herons still have access to food options even in adverse weather conditions or if they are injured. Therefore, while the proximity of water bodies within 0.5 km indicates a preference for wet areas as colony sites, it is essential to acknowledge that these water bodies also serve as primary foraging grounds, given that fish constitute a significant part of the gray heron's diet (Cieślińska et al. 2024). This suggests that the significance of food availability at smaller scales may have been overestimated in this study. The outliers at the 2 km scale indicate that landscape variables at some sampling points differ from those at others. It is hypothesized that once gray herons satisfy their food requirements at smaller scales, they may prioritize other factors over the impacts of additional landscape variables. This idea is supported by findings from previous studies that indicate flexibility in habitat preferences once basic needs are met (Manikowska‐Ślepowrońska et al. 2016), reinforcing the idea that food is a key factor in colony site selection (Atuo and O'Connell 2018; Wamiti et al. 2020). At medium scales, impervious surfaces were initially considered the most important variable, suggesting that gray herons might prefer colony sites associated with a certain amount of human activity. However, our findings imply that impervious surfaces at this scale may be neutral factors when herons breed in areas with sparse buildings (Mauro Fasola and Alieri 1992). This finding implies that gray herons may not be specifically selecting areas with human activity but may also view impervious surfaces in complex landscapes as neutral up to a certain threshold, provided that these surfaces do not pose a significant disturbance. If the disturbance from human activities is within an acceptable range, gray herons may disregard other landscape variables at medium scales. No well‐performing models were observed at larger scales. Overall, regardless of scale, gray herons tend to choose landscapes with complex layouts for their colony sites. However, it is essential to consider the herons' colony and foraging behavior when interpreting the significance of habitat types. The proximity of water bodies within 0.5 km, rather than at distances greater than 10 km, aligns with their preference for wet areas as colony sites and foraging areas within a reasonable commuting distance. This confirms our observation that once gray herons meet their food requirements at smaller scales, they do not significantly weigh the impacts of other landscape variables. Additionally, the significant differences observed in the Shannon diversity index across all distance scales underscore the critical role of landscape complexity in habitat selection by gray herons, aligning with observed patterns in other avian species (Jedlikowski et al. 2016).

### 4.2 Adaptability of Birds to Human Activities

This study highlights that gray herons exhibit flexibility in colony site selection in relation to human disturbance. Our findings indicate that, despite the pressures from urbanization and industrialization, gray herons are capable of adapting their colony strategies to maintain access to suitable habitats. This adaptability suggests a resilience to environmental changes driven by human activities. While pressures from urbanization and industrialization are evident, gray herons adapt their colony strategies in various contexts. This adaptability underscores their resilience to environmental changes, as observed in different regions (Romero‐Vidal et al. 2023; Suvorov and Svobodová 2012). Thus, the claim that gray herons exhibit a general dependency on human activities may be an overstatement, as it varies significantly based on the type of activity and its impact on the habitat. Thus, the claim that gray herons exhibit a general dependency on human activities may be an overstatement, as it varies significantly based on the type of activity and its impact on the habitat.

### 4.3 Model Reliability

In ecological and wildlife habitat selection studies, the presence of outliers often points to anomalous observations in the dataset, which could be due to unusual environmental conditions, data entry errors, or specific behavioral patterns of individual organisms (Canning and Waltham 2021; Haq et al. 2023; Vogel et al. 2022). In this study, identifying and addressing these outliers is crucial for ensuring the accuracy and reliability of the analysis. Gray heron colony site selection might be influenced by various environmental factors, including but not limited to the availability of food resources, habitat types, disturbance from human activities, and other ecological environmental factors (Etezadifar and Barati 2013). The presence of outliers might reflect extreme cases or unique ecological environments within these factors, and their analysis can deepen the understanding of gray herons' responses to complex environmental variables. In this study, the application of the RM model focused not only on the primary environmental variables but also on the identification and treatment of outliers (Alfian et al. 2023; Ijaz, Attique, and Son 2020). By deleting certain sample data and observing changes in model accuracy, we effectively identified outliers that could negatively affect the model's performance. While this method has not yet been widely applied in ecology, it has been utilized in other fields (Christy, Meera Gandhi, and Vaithyasubramanian 2015; Morera et al. 2021). The identification of these outliers not only enhances the reliability of the study but also allows for a better understanding of the factors influencing gray heron colony site selection. This process reflects the adaptability of gray herons to varying environmental conditions and provides important methodological insights for future studies on habitat selection in gray herons and other bird species.

### 4.4 Effectiveness of Cross‐Regional Multiscale Analysis

In recent years, an increasing number of researchers have employed a combination of machine learning methods and land use information to study the landscape characteristics of gray heron colony site selection at larger spatial scales (Carrasco, Mashiko, and Toquenaga 2014; Kelly et al. 2008). Compared to traditional ecological statistical methods such as linear regression, machine learning is more robust in handling complex ecological issues, including multicollinearity and external noise, and is less prone to overfitting (Stock et al. 2018; Stupariu et al. 2022). Additionally, large‐scale analysis can enhance the predictive power and explanatory capacity of models from a macro perspective (Sun et al. 2020). Multiscale integrated analysis combining different spatial scales has become a significant trend and innovation in current research (Compaore et al. 2023; Lin et al. 2023). However, research results using this approach have not yet reached a consensus. For example, Manikowska‐Slepowronska (Jakubas and Manikowska‐Slepowronska 2013), combining GIS analysis and RM model, analyzed the impact of landscape characteristics on the breeding success and colony site selection mechanisms of gray herons in northern Poland. Landscape features within the 0–10 km scale had the highest explanatory power for colony site selection, with the abundance of water bodies and distance from roads being important influencing factors. Carrasco (Carrasco, Mashiko, and Toquenaga 2014) identified medium (4 km) and long (10–15 km) distances as the main factors affecting gray heron colony site selection while studying mixed communities of gray herons and gray heron in the central and southern parts of Ibaraki Prefecture, Japan. At the 4 km scale, evergreen forest areas were particularly important; at the 10–15 km scale, herons tended to avoid urban areas, with food sources not showing significant importance in any of the scale models. On the other hand, a study of gray heron colonies in Yancheng city, Jiangsu Province, China (Wang et al. 2020), considered the 1 km scale features to be the most crucial for explaining colony site selection. These findings highlighted food factors as having a primary influence on colony site choice, partially aligning with our findings. The variability of landscapes among sampling points plays a key role in ecological data analysis using RM model (Mi et al. 2017). As an ensemble learning method, RM improves prediction accuracy and robustness by combining multiple decision trees. Each tree's construction relies on a randomly drawn sample from the entire dataset, and this “bootstrap sampling” method ensures data independence during each tree's training (Breiman 2001). Therefore, landscape variability among sampling points is crucial for ensuring effective training and accurate predictions of the model. If the landscapes of sampling points within a smaller area are highly similar, the lack of sample diversity can lead to a lack of diversity in the decision trees trained by the RM model, reducing the model's generalizability and predictive accuracy for new data (Sun, Peng, and Wu 2022a). Since each tree's training depends on random subsets of samples, if these subsets lack sufficient variability, the model may not effectively capture the key environmental variables and their interactions (Shahhosseini and Hu 2021), thus affecting the model's ability to explain and predict gray heron colony selection behavior. Therefore, inconsistent results across studies do not imply that any are incorrect; models may only have explanatory power in the study area and lack applicability in other regions. To enhance the model's predictive power and applicability, selecting sampling points with sufficient landscape diversity is crucial. This not only aids the RM model in more comprehensively learning and understanding the data but also improves its interpretability for complex ecological data (Wu et al. 2022). The sampling points used in the model cover most regions of China, encompassing diverse ecosystems and environmental conditions from forests and wetlands to deserts. By analyzing data covering a wide range of areas, common patterns in gray heron colony site selection can be identified, as well as unique regional differences. This comparative approach is significant for understanding species distributions and adaptation strategies over large areas.

### 4.5 Research Limitations and Future Directions

Using a combination of the RM model and GIS land use data to explore the landscape characteristics of gray heron colony site selection is an innovative and effective method. The RM model is capable of handling high‐dimensional data and complex data structures, meaning that it can effectively analyze interactions between multiple variables, including those that may influence gray heron colony site selection (Whetten 2021; Zhao and Hou 2022). GIS was utilized in this study to effectively analyze and visualize the geospatial data related to gray heron colony site selection, allowing for a nuanced understanding of habitat preferences across multiple spatial scales. Through GIS, the land use around gray heron colonies, such as water bodies, cropland, and forests (Roccati et al. 2021; Sun et al. 2022b), can be accurately mapped and analyzed, leading to a more precise understanding of the relationship between gray heron colony site selection and the surrounding environment. This study focused mainly on land use types and did not consider other factors, including climatic conditions, that may influence gray heron colony site selection (Borah, Solanki, and Bhattacharjee 2022; Fasola and Cardarelli 2014). The suggestion that migration routes are highly important for gray heron colony site selection is an overstatement (Kumar and Srivastava 2013). While migration routes may influence the breeding range, their specific role in determining local colony site choice requires further investigation (Ye et al. 2018). Analyzing migration routes can help researchers understand how gray herons choose suitable resting and breeding sites during migration. Therefore, future studies should expand the scope to include a variety of environmental factors, including climatic conditions and migration routes. This can be achieved by integrating meteorological data, satellite tracking data, and field survey data. Additionally, employing multimodel integration is also a research trend. Combining the RM model with other ecological models, such as species distribution models (SDMs) and the maximum entropy model (Maxent), can provide a more comprehensive analysis and prediction of gray heron colony site selection.

In summary, the RM model and GIS were applied to study the landscape characteristics of gray heron colony site selection in China, yielding new findings that gray herons exhibit a certain flexibility in their colony t site selection. This indicates that the availability of food resources and a complex landscape layout are potential conditions for their colony site selection, which is a significant conclusion in explaining their widespread global distribution. Additionally, the conclusions suggest that gray herons demonstrate a degree of tolerance to human activities, offering valuable insights into conservation strategies for this species. This implies that effective protection may be achieved by minimizing disturbances. Finally, the importance of interdisciplinary collaboration is highlighted, combining knowledge from fields such as ecology, geography, and climatology to provide a more comprehensive perspective and in‐depth analysis for the conservation of wildlife.

## 5 Conclusions

This study advances our understanding of gray heron colony site selection through the use of GIS and random forest models. By exploring cross‐regional and multi‐scale influences, we found that gray herons adapt flexibly to environmental conditions, particularly foraging habitats and human disturbances. These findings are crucial for informing conservation strategies aimed at minimizing disturbances near key habitats. Moreover, the methods developed here can be applied to other avian species, potentially contributing to broader conservation efforts. Future studies could integrate additional ecological and environmental data for more precise predictions of habitat selection.

## Author Contributions

**Ran Tian:** conceptualization (equal), data curation (equal), formal analysis (equal), investigation (equal), methodology (equal), resources (equal), software (equal), supervision (equal), validation (equal), visualization (equal), writing – original draft (equal), writing – review and editing (equal). **Yongbin Zhao:** conceptualization (equal), formal analysis (equal), funding acquisition (equal), project administration (equal), resources (equal), software (equal), supervision (equal), writing – original draft (equal), writing – review and editing (equal). **Donghong Li:** data curation (equal), formal analysis (equal), writing – review and editing (equal). **Shiyu Zhang:** data curation (equal), formal analysis (equal), writing – review and editing (equal). **Guodong Yi:** data curation (equal), formal analysis (equal), writing – review and editing (equal).

## Conflicts of Interest

The authors declare no conflicts of interest.

## Open Research Statement

The land use information tif file comes from Earth System Science Data, DOI: 10.5194/essd‐13‐3907‐2021.

Pseudoabsence points comes from GBIF (https://www.gbif.org/).

In this study,16 out of 19 sample points were obtained from CNKI (https://kns.cnki.net/kns8s/), DOI: 10.13248/j.cnki.wetlandsci.2021.05.009; 10.13248/j.cnki.wetlandsci.2016.02.014; 10.13448/j.cnki.jalre.2015.167; 10.19711/j.cnki.issn2310‐1490.2008.01.008; 10.3969/j.issn.1006‐6993.2015.01.039; 10.19711/j.cnki.issn2310‐1490.1990.03.010; 10.3969/j.issn.1005‐4707.2012.03.025; 10.13859/j.cjz.2002.02.020; 10.13859/j.cjz.2008.06.001; 10.13989/j.cnki.0517‐6611.2011.28.012; 10.19711/j.cnki.issn2310‐1490.2013.04.004; CNKI:SUN:SCDW.0.2019‐02‐018; 10.19711/j.cnki.issn2310‐1490.2012.03.009; 10.16202/j.cnki.tnrs.1989.03.014; 10.3969/j.issn.1000‐7083.2002.02.014.2 out of 19 sample points were obtained from cqVIP (https://qikan.cqvip.com/), DOI: 10.5122/cbirds.2010.0010; 10.7666/d.y1996900. Two out of 19 sample points were obtained from Wanfang (https://www.wanfangdata.com.cn/index.html), CNKI:SUN:YSDW.0.1997‐04‐013; 10.3969/j.issn.1001‐1714.2002.01.005. The other two sample points in this study were from field investigations. The 79 colony sites obtained from the GBIF website (Locations where both gray herons and colonies appear in the same photograph are considered gray heron colony sites.)

## Supporting information




Additional file 1‐10

## Data Availability

The data that support the findings of this study are available in [Zenodo] at [https://zenodo.org/], reference number [4417810]. These data were derived from the following resources available in the public domain: [https://kns.cnki.net/kns8s/] [https://qikan.cqvip.com/] [https://www.wanfangdata.com.cn/index.html] [https://www.gbif.org/].
