# Supplementary figures and images for: Colony Site Selection of Gray Heron (Ardea cinerea) During the Breeding Period at Multiple Spatial Scales
Source: Ecol Evol. 2025 Feb 12;15(2):e70937. doi: 10.1002/ece3.70937 (PMC11814540; doi:10.1002/ece3.70937)

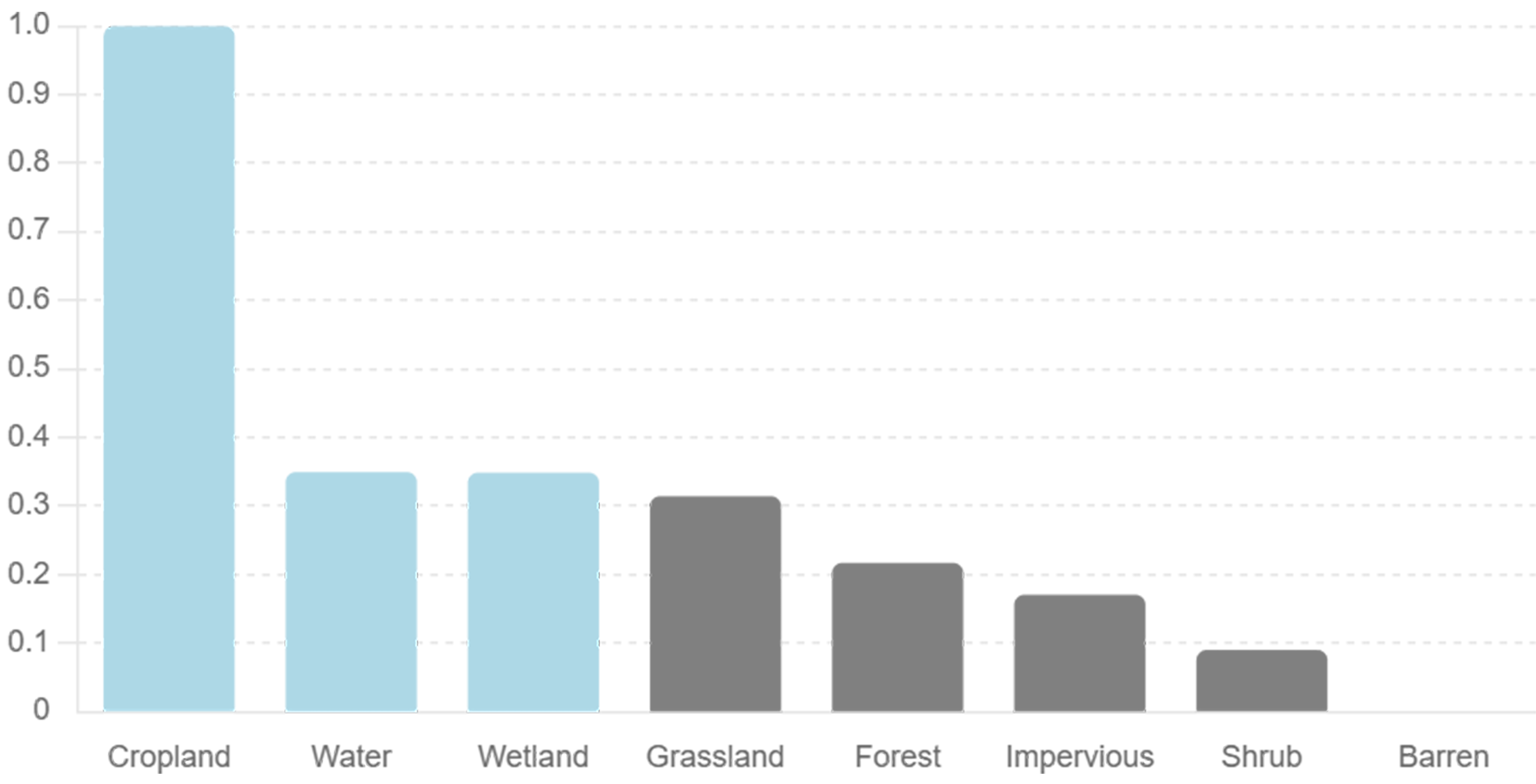

Supplement: Supplementary file 1 — Additional file 1‐10 [file ECE3-15-e70937-s001.zip › ece370937-sup-0001-supinfo/ece370937-sup-0001.tiff]

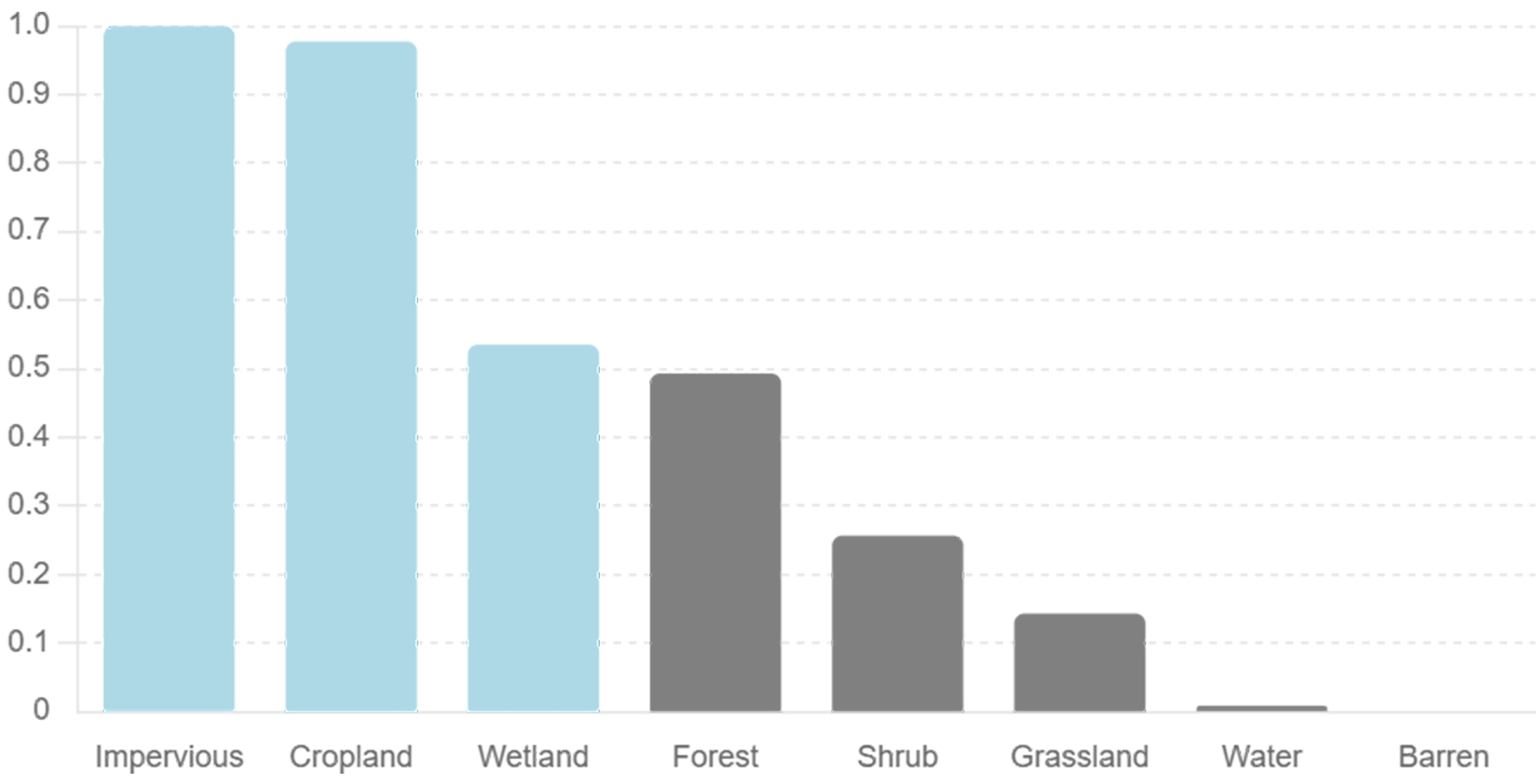

Supplement: Supplementary file 1 — Additional file 1‐10 [file ECE3-15-e70937-s001.zip › ece370937-sup-0001-supinfo/ece370937-sup-0002.tiff]

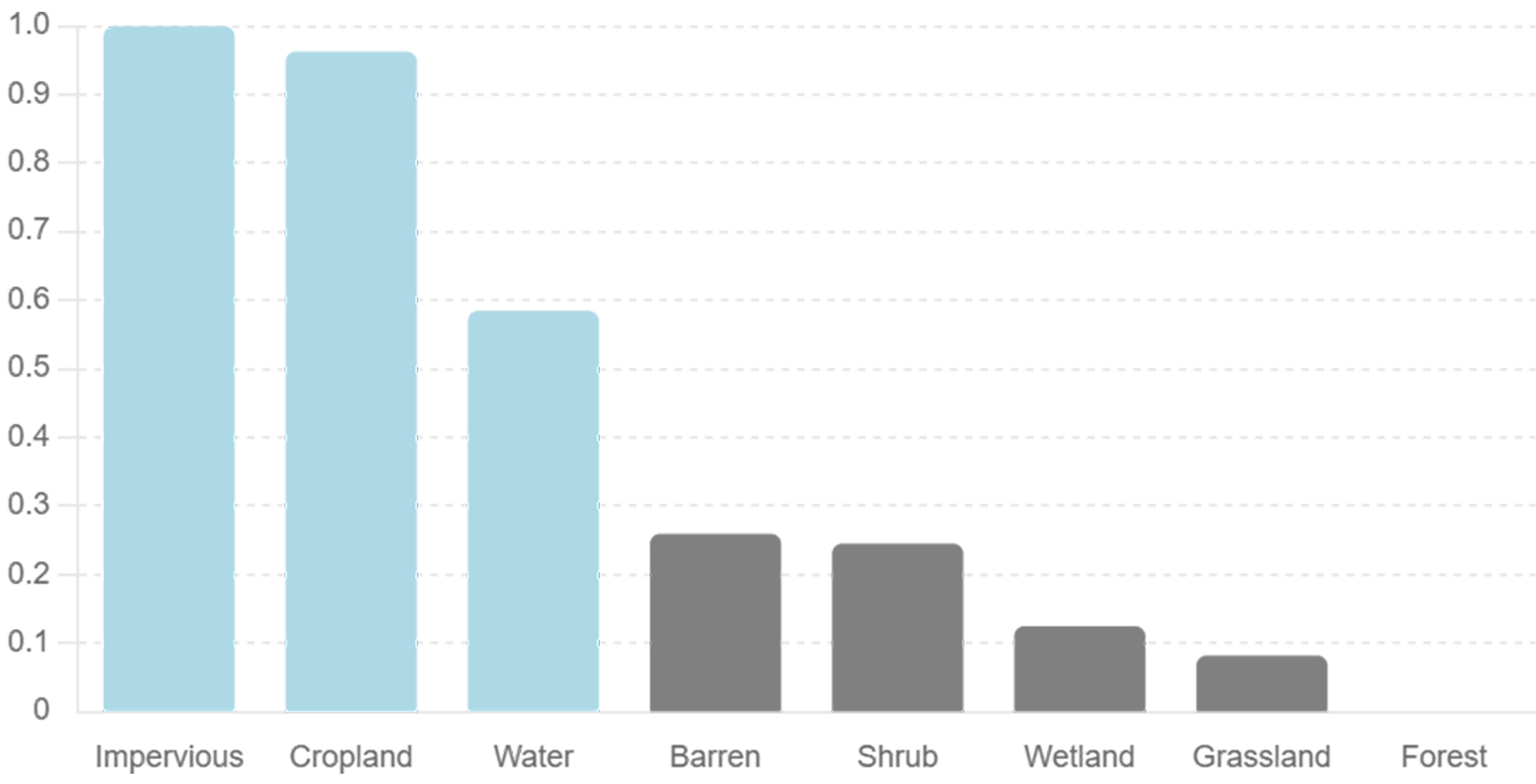

Supplement: Supplementary file 1 — Additional file 1‐10 [file ECE3-15-e70937-s001.zip › ece370937-sup-0001-supinfo/ece370937-sup-0003.tiff]

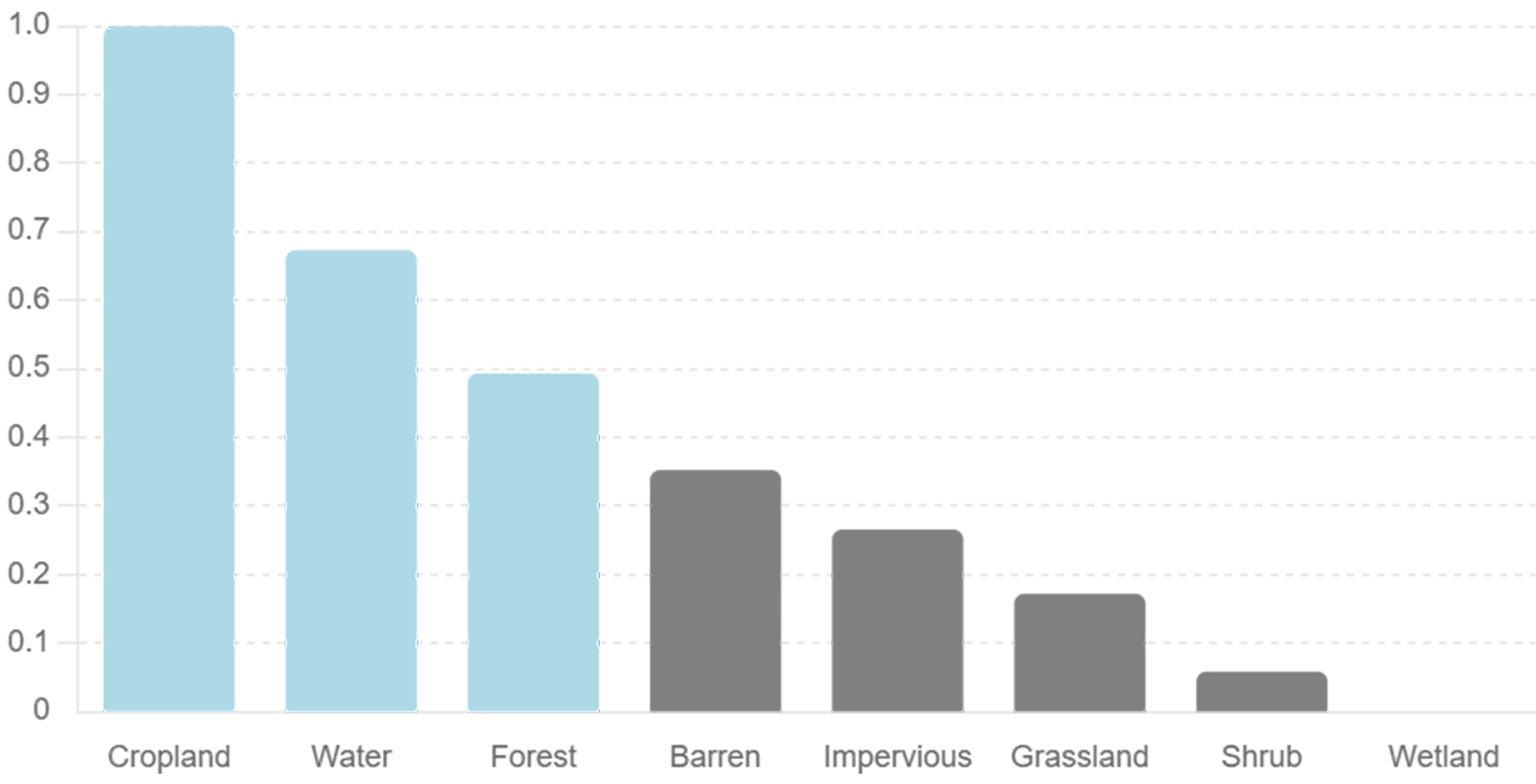

Supplement: Supplementary file 1 — Additional file 1‐10 [file ECE3-15-e70937-s001.zip › ece370937-sup-0001-supinfo/ece370937-sup-0004.tiff]

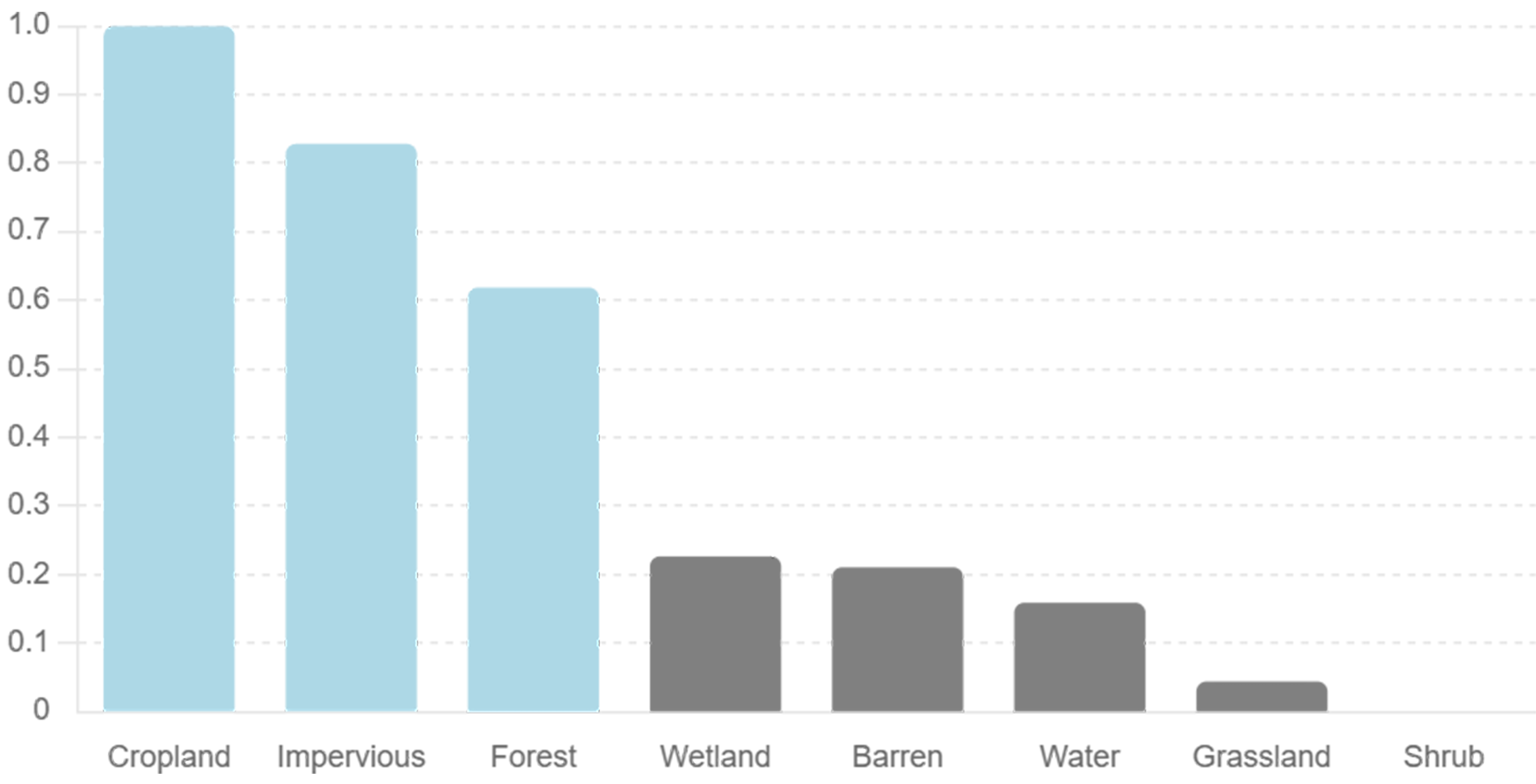

Supplement: Supplementary file 1 — Additional file 1‐10 [file ECE3-15-e70937-s001.zip › ece370937-sup-0001-supinfo/ece370937-sup-0005.tiff]

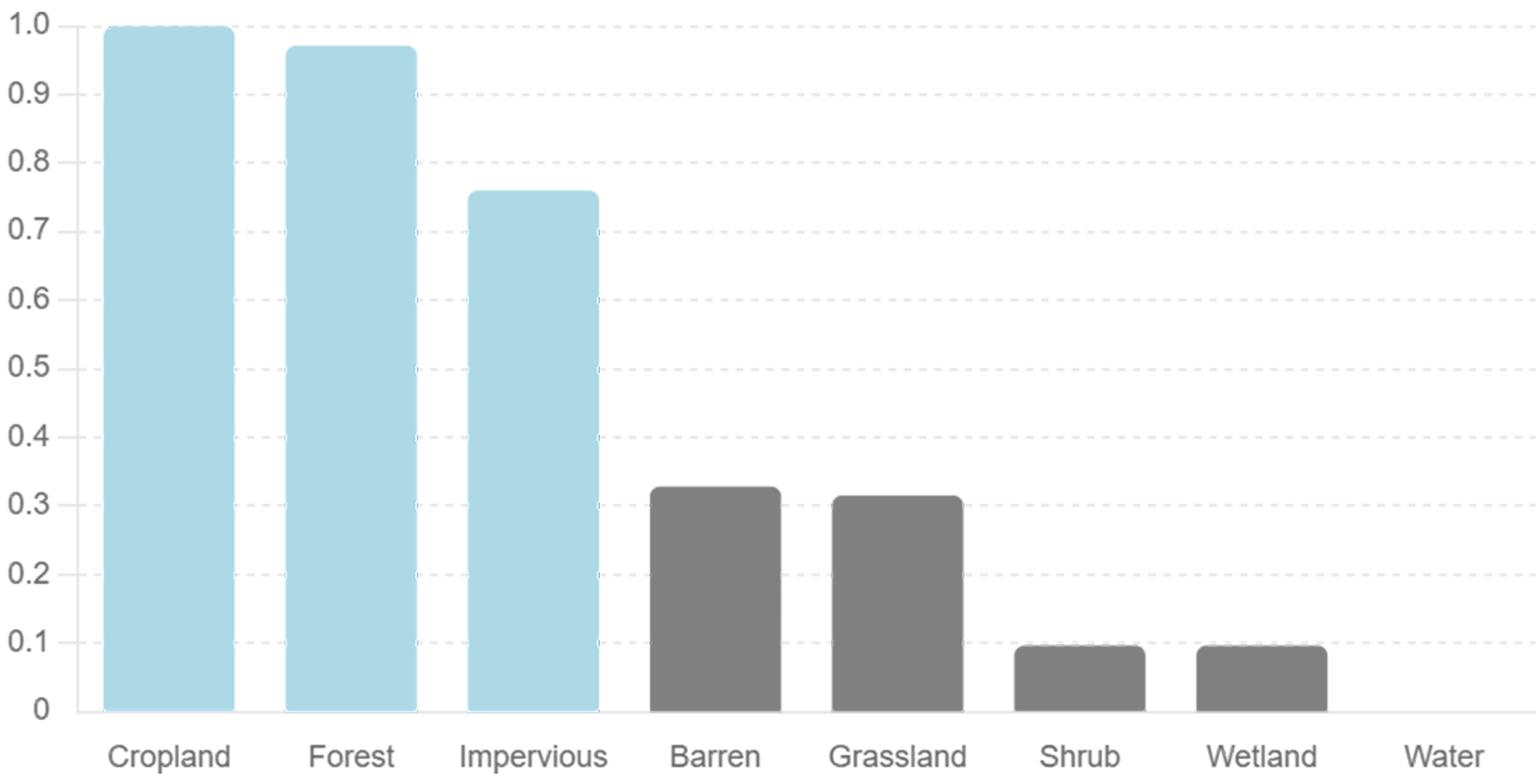

Supplement: Supplementary file 1 — Additional file 1‐10 [file ECE3-15-e70937-s001.zip › ece370937-sup-0001-supinfo/ece370937-sup-0006.tiff]

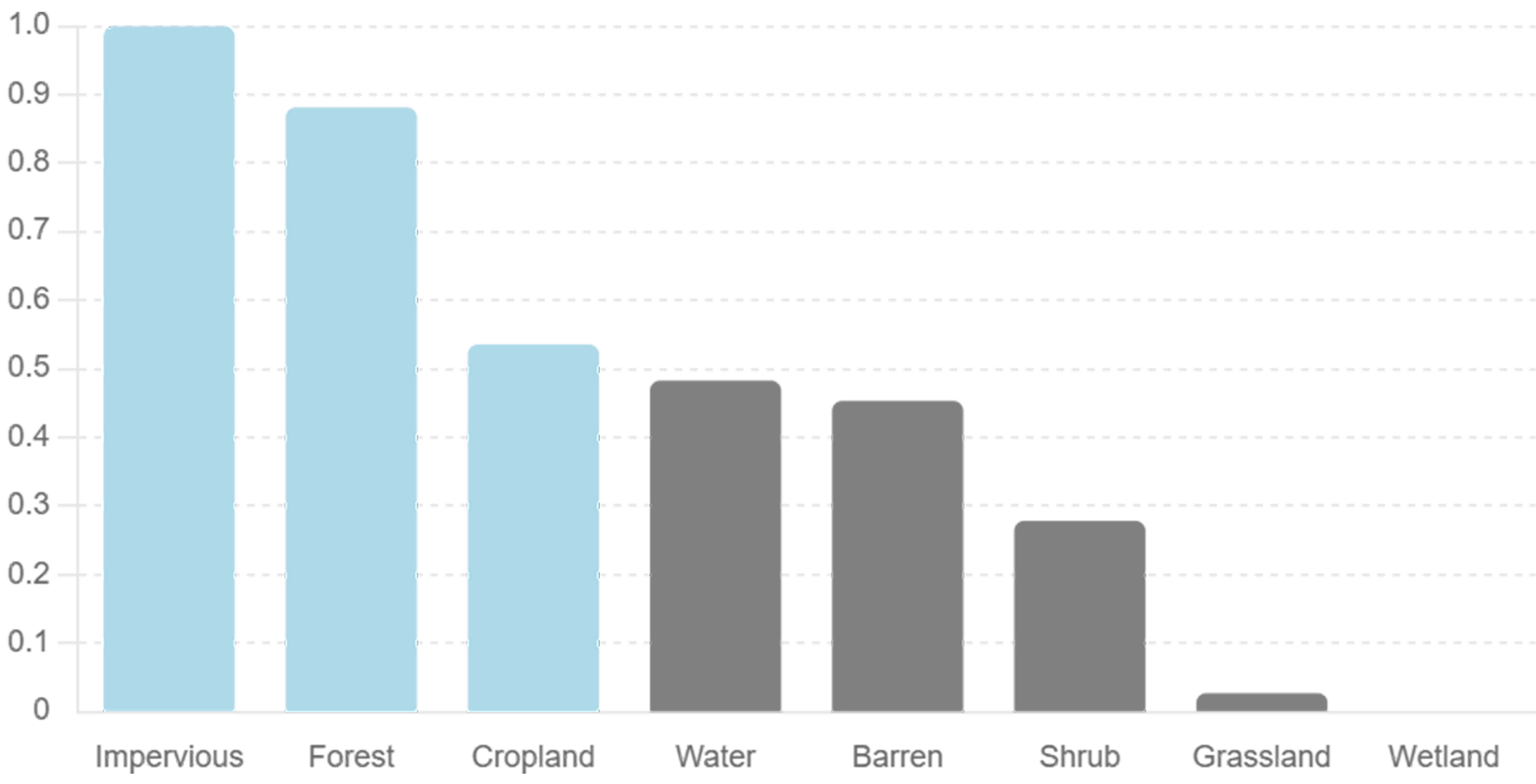

Supplement: Supplementary file 1 — Additional file 1‐10 [file ECE3-15-e70937-s001.zip › ece370937-sup-0001-supinfo/ece370937-sup-0007.tiff]

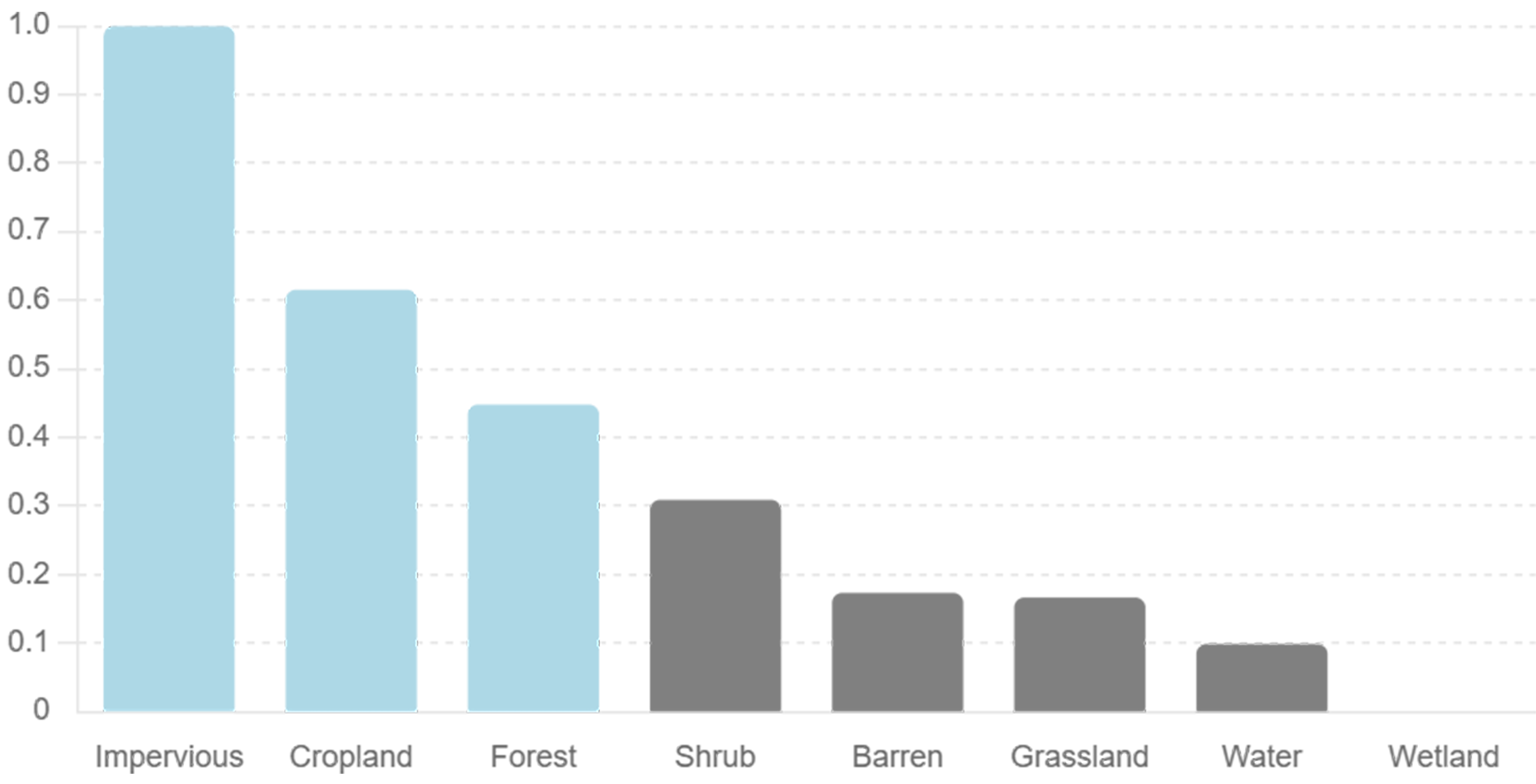

Supplement: Supplementary file 1 — Additional file 1‐10 [file ECE3-15-e70937-s001.zip › ece370937-sup-0001-supinfo/ece370937-sup-0008.tiff]

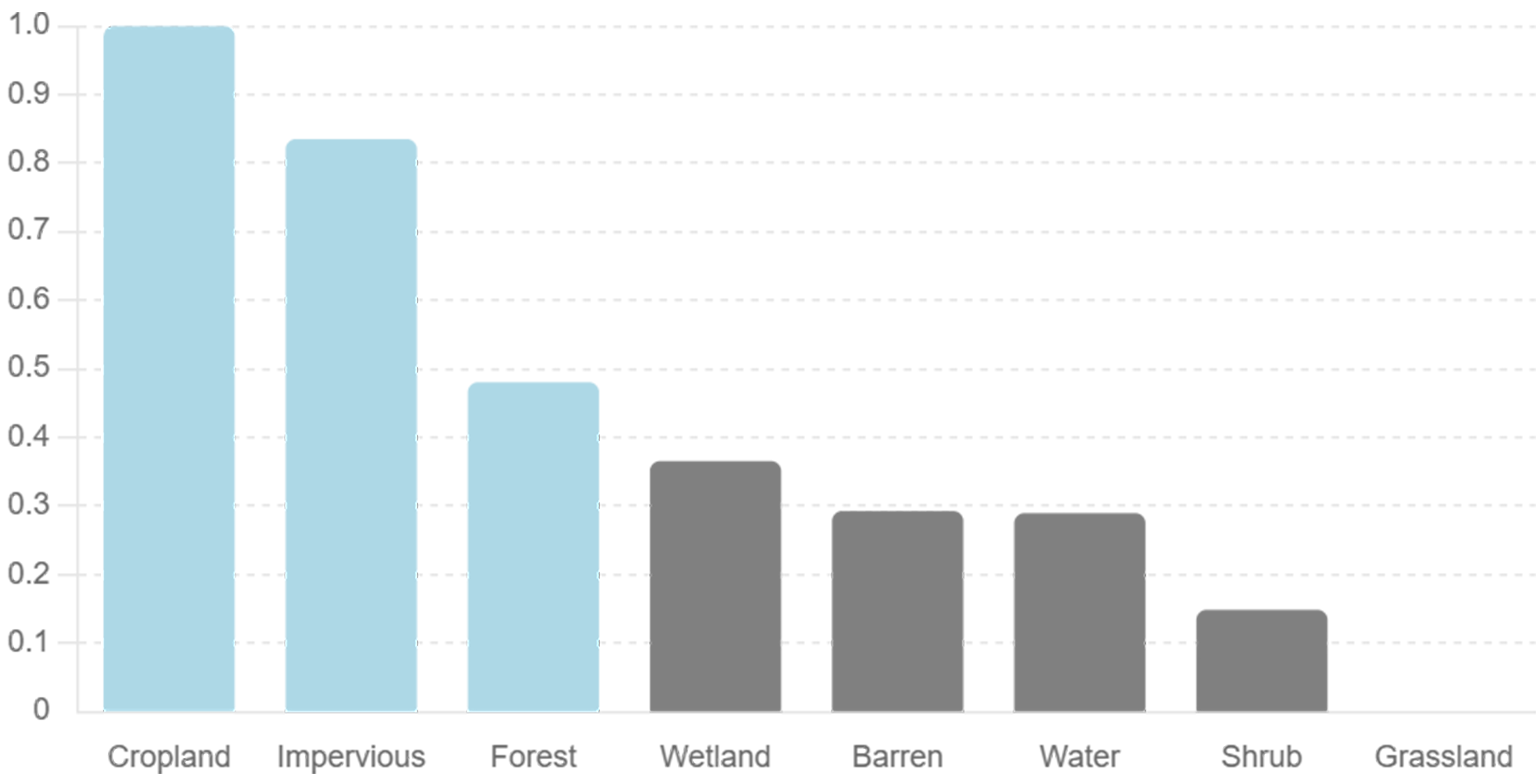

Supplement: Supplementary file 1 — Additional file 1‐10 [file ECE3-15-e70937-s001.zip › ece370937-sup-0001-supinfo/ece370937-sup-0009.tiff]

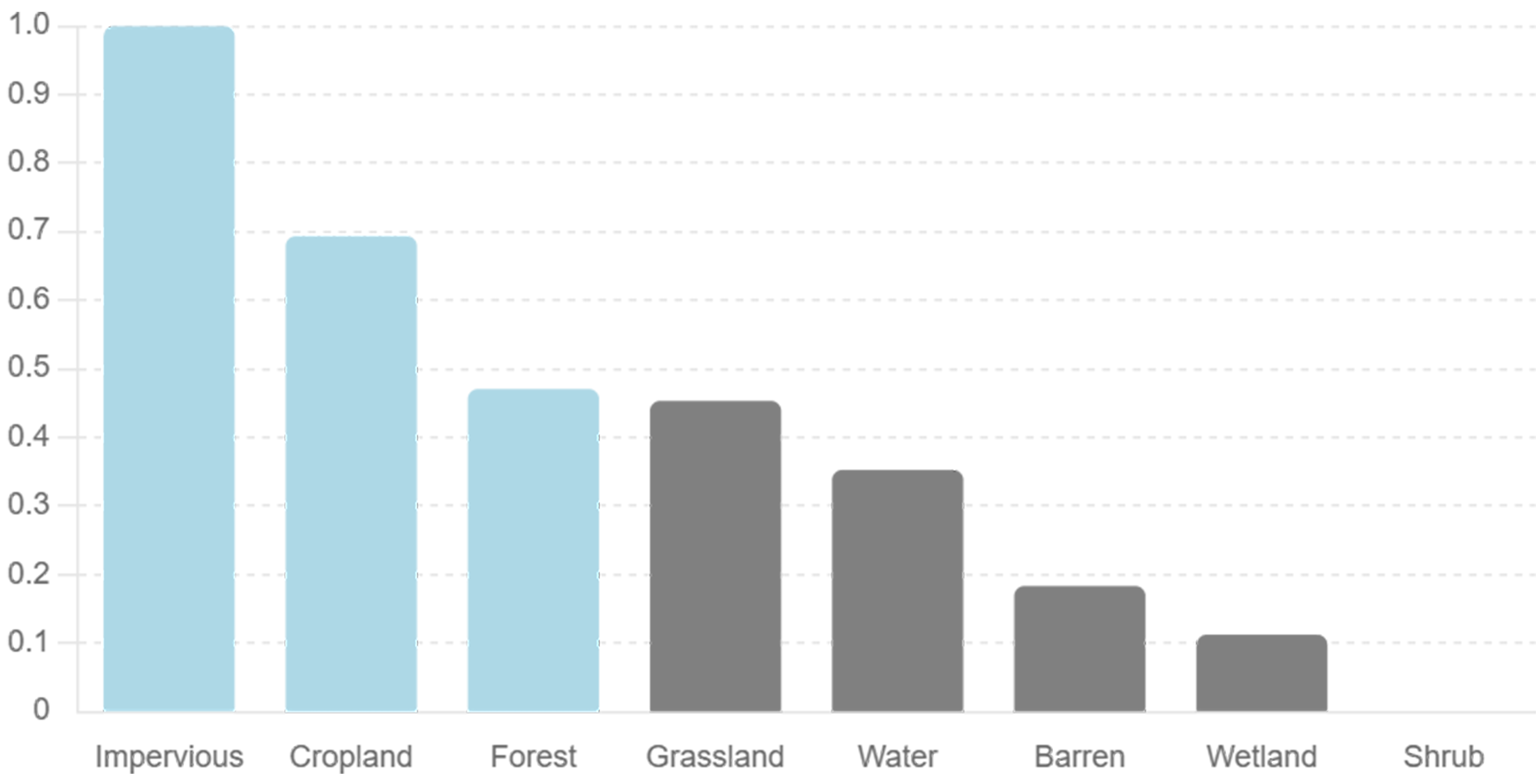

Supplement: Supplementary file 1 — Additional file 1‐10 [file ECE3-15-e70937-s001.zip › ece370937-sup-0001-supinfo/ece370937-sup-0010.tiff]
